# Surface-dependent scenarios for dissolution-driven motion of growing droplets

**DOI:** 10.1038/s41598-017-00886-2

**Published:** 2017-04-19

**Authors:** Stefano Curiotto, Frédéric Leroy, Fabien Cheynis, Pierre Müller

**Affiliations:** Aix Marseille Univ, CNRS, CINaM, Marseille, France

## Abstract

Nano-droplets on a foreign substrate have received increasing attention because of their technological possible applications, for instance to catalyse the growth of nanowires. In some cases the droplets can move as a result of a reaction with the substrate. In this work we show that the substrate orientation, the surface morphology and the shape of the pits etched in the substrate by the droplets affect the droplet motion, so that a single mechanism (droplet-induced substrate dissolution) may lead to several unexpected droplet dynamics. The experiments are carried out by low energy electron microscopy on Au-Si and Au-Ge, which are model systems for studying liquid droplet alloys. Studying *in-situ* the behaviour of Au droplets on various Si and Ge surfaces, we describe a subtle interplay between the substrate orientation, the surface defects, and the droplet motion. Our observations allow a deep understanding of the interfacial mechanisms at the origin of the alloy formation and the associated droplet motion. These mechanisms are based on events of substrate dissolution/recrystallization. The outcomes of this work highlight the importance of the etching anisotropy on the droplet-substrate behaviours, and are essential in the perspective of positioning liquid alloy droplets used for instance as nanowire catalysts.

## Introduction

**Figure 1:**
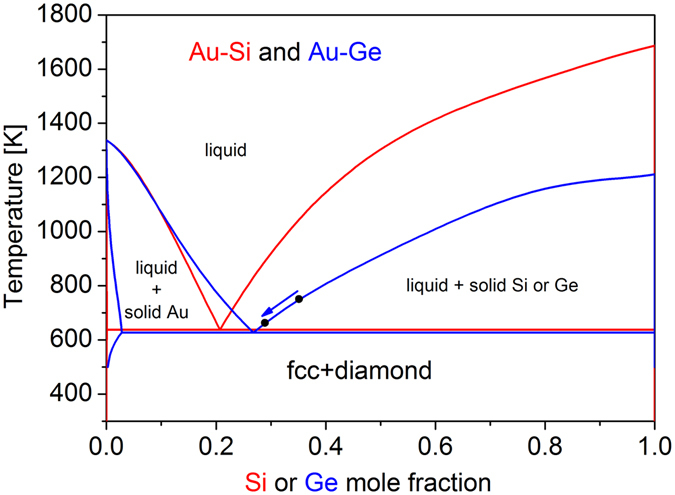
Au-Si (red) and Au-Ge (blue) phase diagrams of the bulk phases, calculated using the thermodynamic optimizations of Meng *et al*.^44^ (Au-Si) and Chevalier^45^ (Au-Ge). The arrow shows the composition change in a Au-Ge droplet on decreasing temperature, as discussed in the text.

The motion of small particles on a surface has recently attracted the interest of the scientific community. Several different driving forces have been identified as for instance Marangoni effect^1^, electromigration^2–5^, non congruent evaporation^6–8^, wettability gradients^9, 10^, and chemical reactivity^6, 11, 12^. In this last case, the reaction between a particle and its underlying substrate generally leads to a local modification of the interface at the origin of a spontaneous motion. For a given reaction between a particle and a substrate, the particle motion actually depends on the surface orientation as well as on the surface morphology. Though evidenced by a few experimental results, there is no systematic study (for a given couple of materials) of the influence of these parameters on the particle behaviour. The control of the motion of self-propelled particles requires understanding of local effects like dissolution or pinning. In this work we report on the self-propulsion of particles that form a eutectic alloy with their underlying substrate. More precisely, if a solid particle of one material is deposited on another material and annealed above the bulk eutectic temperature of the binary system, the particle transforms into a liquid alloy. This implies that the substrate is locally dissolved and provides atoms to reach the eutectic composition inside the droplet. Due to crystalline anisotropy, the dissolution kinetics depends on the crystallographic orientation of the substrate surface. If interfacial dissolution occurs fast, the particle may simply etch locally the underlying substrate on spot to reach the equilibrium composition. On the contrary, if interfacial dissolution is slow, the droplet might not reach easily the equilibrium composition. However, if the surface offers local heterogeneities easier to dissolve, this deviation from equilibrium may provide a driving force for the droplet motion. In this work we have studied the case of Au/Si and Au/Ge alloys. The main advantages of these systems are that they both form a eutectic at a temperature *T*_*E*_ ≈ 630 K (see Fig. 1) and present a strong anisotropy of dissolution kinetics^13, 14^. Besides, the two systems have a technological interest, as metallic droplets on semiconductor substrates catalyze the growth of nanowires by the Vapor Liquid Solid method^15^ and present features similar to those of III-V nanowires, which are widely investigated (for a review see ref. 16).

## Dissolution Anisotropy

**Figure 2:**
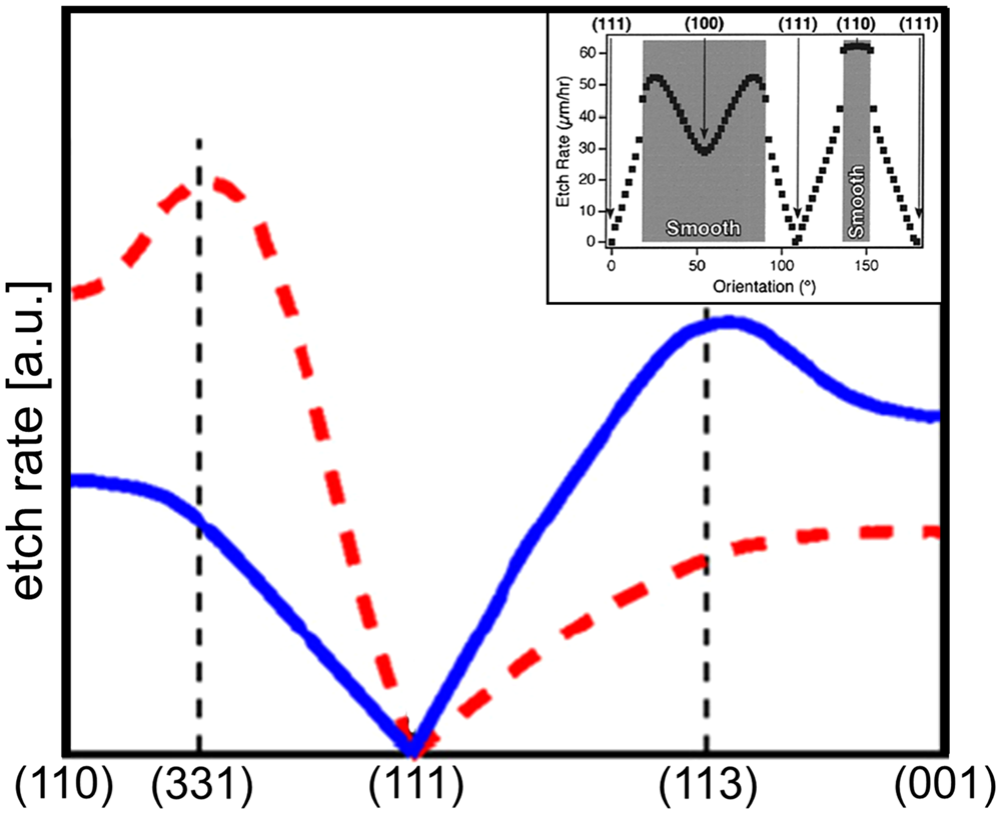
Si etch rate under low (blue) and high (red) reactant concentration, redrawn from ref. 14. The graph gives information on the Si dissolution anisotropy. Inset: Experimental etch rate of different Si crystallographic orientation by wet etching (KOH), reprinted (adapted) with permission from^23^. Copyright 2002 American Chemical Society.

Dissolution of Si and Ge by various etchants has been widely studied since the seventies^17^. The anisotropy of dissolution of Si and Ge has been the subject of several works due to the wide use of chemical etching of Si and Ge in the semiconductor industry^13, 18–22^. The etching rates depend on the type of reactant and its adsorption properties at the surface, but the dissolution anisotropy depends essentially on the crystallography. Since {111} surfaces are denser, the atoms are more bound and therefore harder to dissolve than on other surfaces. Figure 2 shows a typical variation of the Si dissolution rate between (111), (110) and (100) orientations calculated at low (blue) and high (red) concentration of reactants^14^ with, in the inset, an example of experimental etching rates measured in alcaline solution^23^. The dissolution of Ge is less documented but (i) the etching anisotropy of Ge seems to be weaker than that of Si (at least in basic solutions)^13^; (ii) the hierarchy of etching rate for the different orientations is equal to that of Si; (iii) due to the low cohesion energy of Ge compared to Si, the Ge dissolution rates are generally higher than those of Si^18, 20, 21^ (for completeness notice that Ge may be more rapidly etched than Si when using plasma dry etching under specific conditions^24^).

The shape of growing droplets is controlled by the underlying substrate symmetry because they are generally limited by the edges of the anisotropic etch pit that they have dug in the substrate. Therefore the top view of the droplets on the surface appears square, or elongated on (100) and (110) surfaces respectively, while on (111) triangular droplets are occasionally observed^25^.

**Figure 3:**
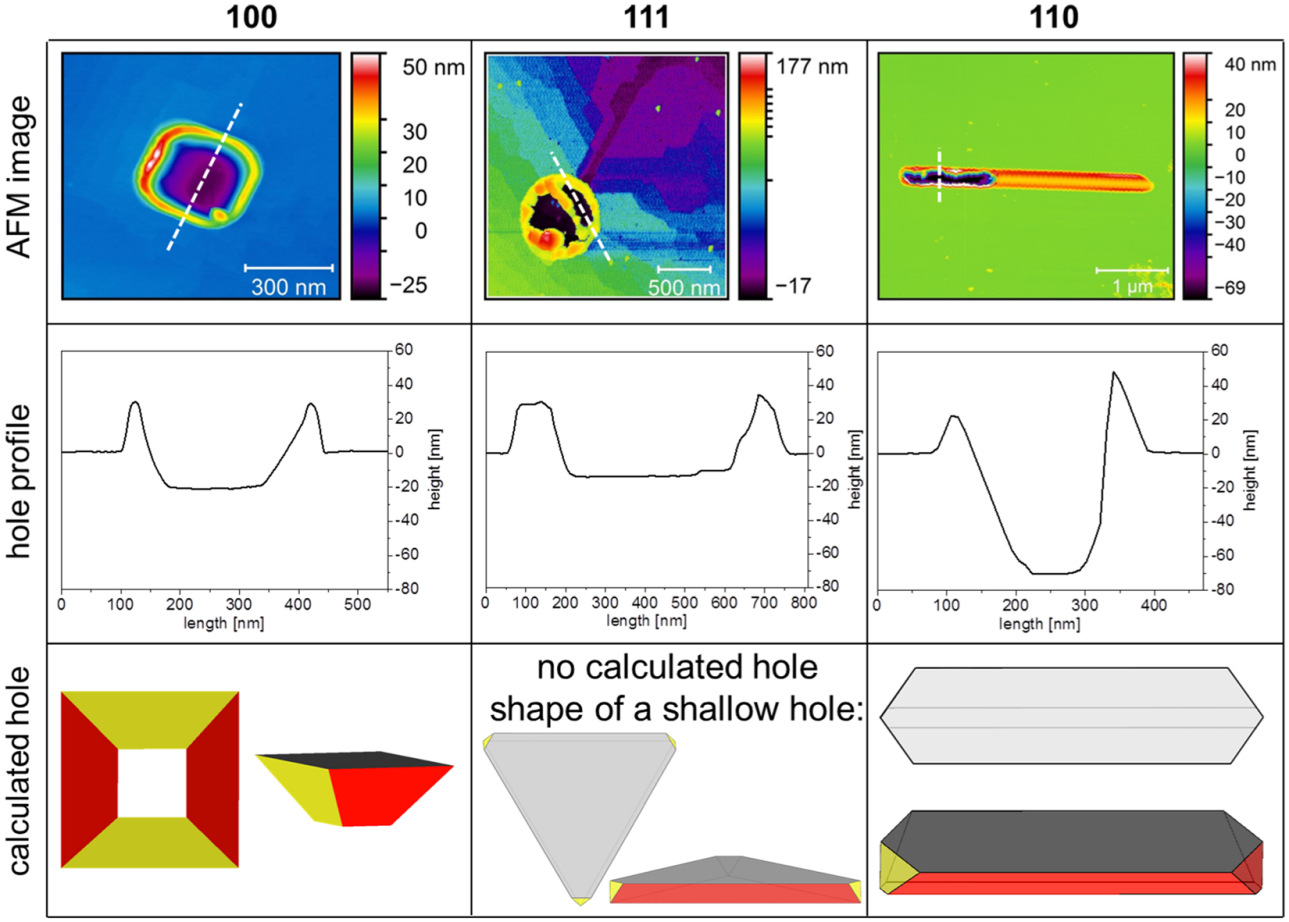
Etch pits due to interfacial reaction of Au on Si substrates with different crystallographic orientations. The first row shows three AFM images of the holes left by droplets on substrates (100), (111) and (110). The second row shows the AFM height profiles taken along the white dashed lines of the AFM images. Si dissolved when the droplets were liquid is preferentially redeposited, on solidification, at the edges of the holes; for (111) substrates it is also visible in the middle of the holes (after Au etching). The third row shows the calculated shapes of holes limited by {111} facets in the different Si substrates.

AFM observations of the pits have been done after Au removal from the samples by KI/I_2_ etching (see methods). Figure 3 summarizes the shape of different etch pits left by the dissolution of the Si substrate to form Au-Si alloy droplets. Similar results have been obtained for Au droplets on Ge surfaces by Jung *et al*.^22^. The figure shows AFM images of the etch pits (first line), etch pit profiles (second line) and the shape of the droplet-substrate interface calculated in the case of dissolution limited by {111} planes, for {100}, {111} and {110} orientations. On the (100) surface, a Au droplet etches the underlying substrate, forming a deep hole limited by {111} lateral facets. Our findings are in agreement with the results of Nikiel *et al*.^26^ and Rath *et al*.^27^, who have shown the formation of holes under Au particles formed by dewetting of thin Au films on Ge(100). On (111) surfaces a droplet leaves a track which extends far behind the droplet (violet part of the AFM image in Fig. 3 and see later in the text). As described in ref. 25, the droplet moves at the surface and nibbles the ascending monoatomic steps encountered during motion. On (110) the droplet is elongated and thus leaves an elongated etch pit, but, surprisingly, also leaves a Si nanowire behind^28^. Notice that the volume of the etch pit left in the (100) substrate roughly corresponds to the amount of material that must be dissolved to reach the Au-Si alloy composition in the droplet. However, this is not the case neither for Si(111), where the etch pit is very shallow (as expected for a surface that is hard to dissolve), nor for Si(110), where the volume of the etch pit is larger than the volume of the emerging part of the droplet. As discussed later, this over-etching is at the origin of the nanowire formation.

## Droplet motion on (111) surfaces

AFM experiments (Fig. 3) show that, while for easy interfacial dissolution, as (001) surfaces, a growing droplet simply dissolves its underlying substrate on the spot, it is not the case on surface orientations harder to dissolve (see Fig. 2), like (111) surfaces, where droplet motion is favored to dissolve the substrate atoms from less bound sites. Therefore, the local environment of the droplet, and in particular surface defects, plays an important role on the movement. Different scenarios have been observed according to the local geometry close to the droplet:

1. A droplet nucleates at the edge of a 2D substrate island, nibbles it and remains attached to the 2D shrinking island edge.
2. A droplet nucleates on a monoatomic step, preferentially dissolves the step and thus moves along it.
3. A droplet makes a dent in a step, then it continues to dissolve atoms from the dent. The droplet displaces across the terrace and forms a track behind.
4. On vicinal surfaces, a droplet may stride across several steps and thus climb up the steps by local dissolution leaving behind it a deep track formed by the notched steps.

**Figure 4:**
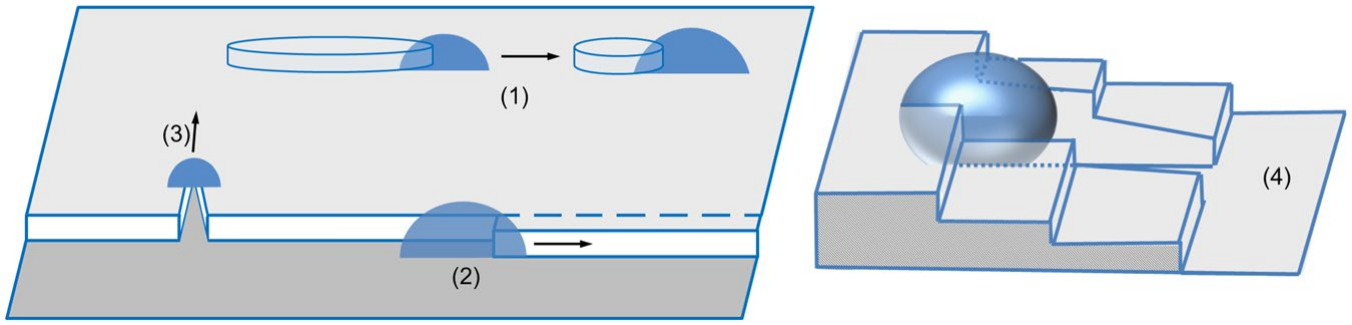
Scenarios of droplet motion on a (111) surface, as explained in the text.

These scenarios are sketched in Fig. 4 and discussed in the following sections.

### 2D islands as a reservoir for droplets

**Figure 5:**
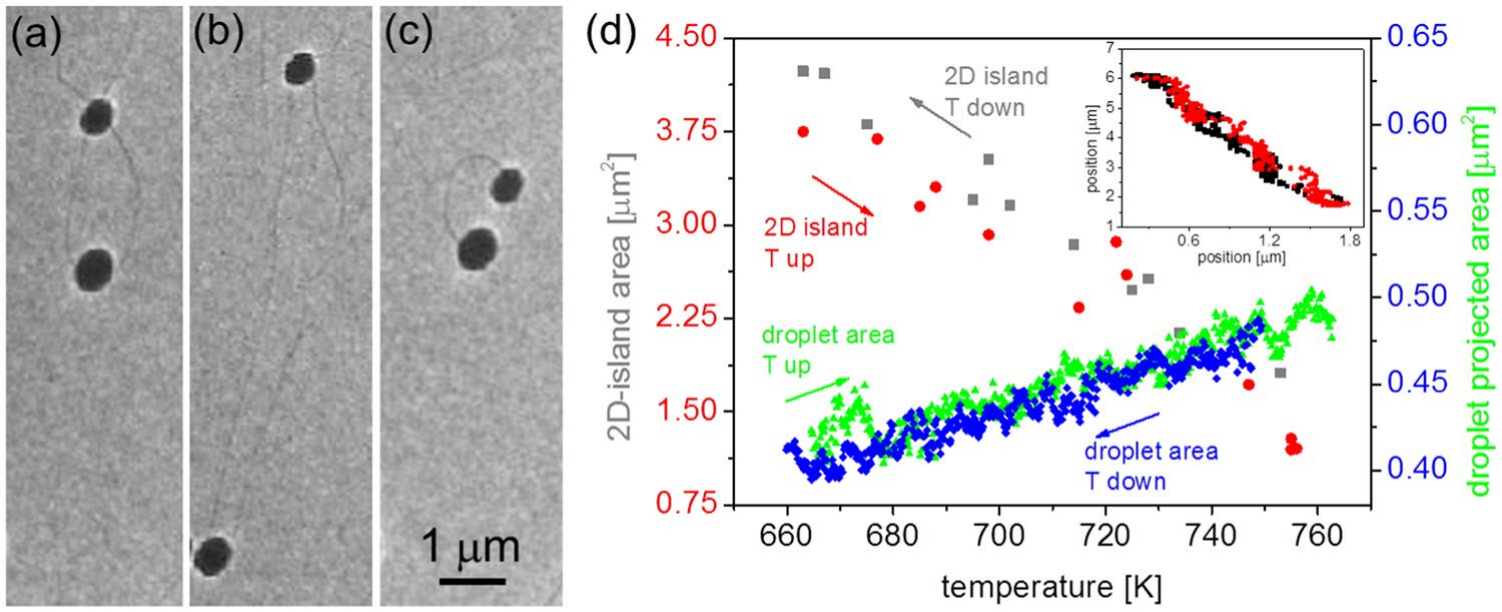
(**a**–**c**) LEEM images (E = 2 eV) of two droplets on Ge(111) after Au deposition (~4 · 10^19^ at · m^−2^). The sample temperature is decreased from 750 K (**a**) to 660 K (**b**), and then increased back to 750 K (**c**) (heating and cooling rate = 7 K/minute); (**d**) Change of the total droplet projected area (right axis, blue diamonds for the temperature decrease and green triangles for the temperature increase) and of the 2D island area (left axis, grey squares for the temperature decrease and red circles for the temperature increase). Inset: position of the lower 3D droplet during temperature decrease (black) and increase (red).

In this section we show that the droplet motion is associated to the substrate dissolution. A temperature variation changes the alloy equilibrium concentration and induces the droplet motion. Figure 5 shows two droplets at the edge of a 2D Ge island. Decreasing the temperature from 750 K to 660 K, the droplet size decreases but the area of the 2D island increases (see also Supplementary Video S1). On the contrary, increasing the temperature back to 750 K, the droplet size increases again and the 2D area decreases. Since the droplets are pinned at the 2D island edge, they are dragged by the edge motion associated to the 2D area change. Decreasing the temperature, the droplets become supersaturated in Ge and thus release Ge to keep their equilibrium concentration (see the arrow in Fig. 1). The amount of released Ge is transferred to the 2D island which thus enlarges. Increasing the temperature, the reverse occurs, the droplets become undersaturated and thus incorporate Ge from the 2D island. Some authors have observed the formation of metastable Au-Ge phases in rapidly solidified alloys or undercooled nanodroplets (see as examples refs 29 and 30). In our case, the formation of metastable phases on cooling is unlikely because the cooling rate is slow (7 K/minute) and the minimum temperature is at least 25 K above the eutectic. The interplay between the 2D island and the droplets is illustrated in Fig. 5 by the reversibility of the size variation of both droplets (green triangles and blue diamonds) and 2D island (red circles and grey squares) with temperature.

**Figure 6:**
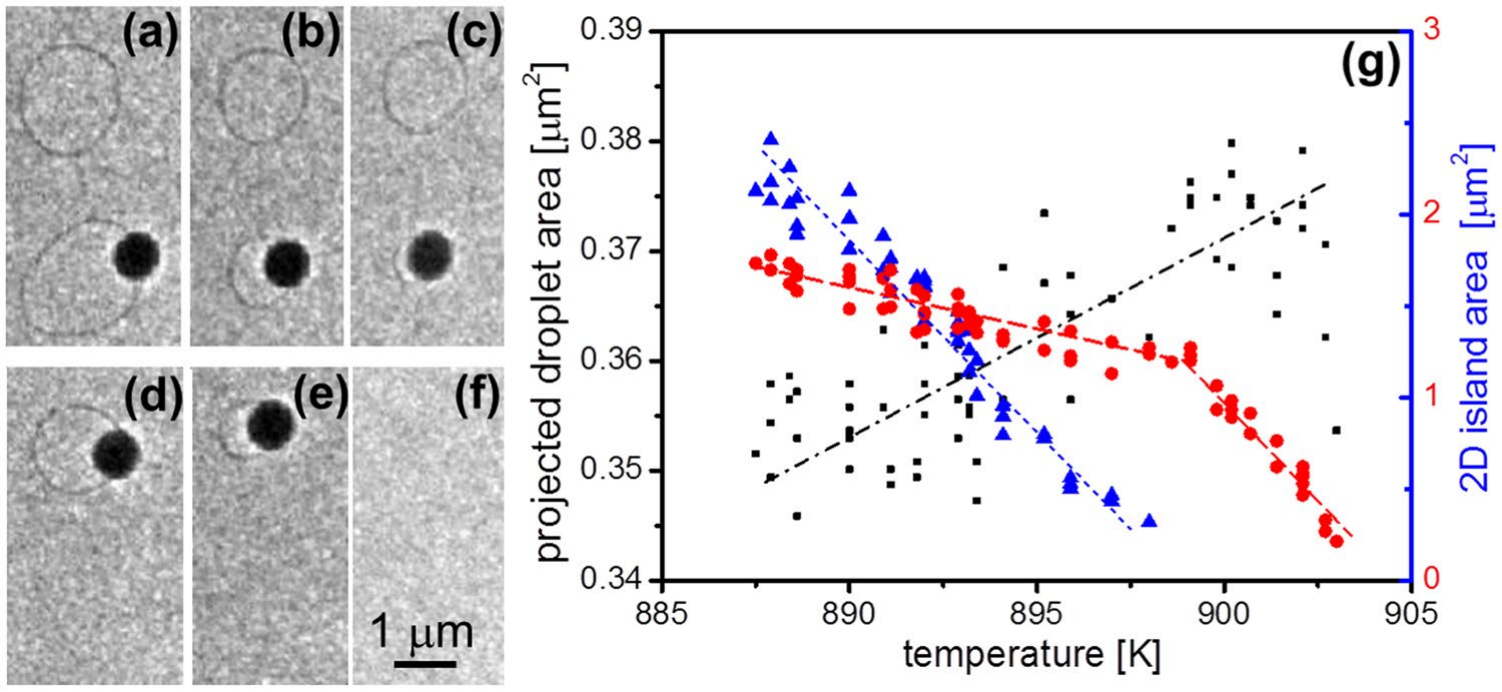
(**a**–**f**) LEEM images (E = 2 eV) of a droplet on Ge(111) after Au deposition (~3 · 10^19^ at · m^−2^). The droplet consumes a 2D island (**a**–**c**) before jumping to another one (**d**,**e**) and consuming it; the temperature increases from 890 K (**a**) to 900 K (**f**). (**g**) Left axis: projected droplet area (black squares); right axis: area of the 2D island in the upper part of images (**a–f**) (red circles) and of the island in the lower part (blue triangles). Lines are only guide for the eyes but clearly show the slope breaking (red) due to the droplet effect.

From an experimental point of view it is difficult to go beyond this semi-quantitative study of the interplay between a 2D island and a droplet because droplets and islands behave differently according to their environment. In fact, other phenomena like pinning at defects, Gibbs-Thomson effect, Ostwald ripening or sublimation (for the higher temperatures) can occur. Thus, a careful quantitative study would require to decouple these effects from the droplet motion due to substrate dissolution. In some cases this is possible, as for the droplet of Fig. 6 where two 2D islands coexist at a close distance (see also Supplementary Video S2). The upper one is a free 2D island whereas the lower one is associated to a droplet. Increasing the temperature, both 2D islands shrink but for different reasons. The area of the upper island essentially decreases by its own (self-shrinking due to Gibbs-Thomson effect, Ostwald ripening or sublimation) while the lower 2D island is also consumed by the droplet. The shrinking rate of the lower island is larger than that of the upper island (Fig. 6b). When the lower 2D island has been completely consumed, the droplet is no more pinned and can thus move on the surface. In the studied case the droplet moves along a surface domain boundary towards the edge of the upper island where it pins again. The 2D island now shrinks more rapidly since it is not only consumed by self-shrinking but also by dissolution of Ge in the droplet. Since both 2D islands have similar sizes, this experiment enables to discriminate the self-shrinking effect from the 2D island/droplet interplay. The volume of the droplet can be calculated from the projected area, assuming the droplet is a spherical cap with contact angle *θ*. The volume change gives the number of atoms incorporated in the droplet because of substrate dissolution: ${\rm{\Delta }}{V}_{droplet}={\rm{\Delta }}{A}_{droplet}^{{\rm{3 / 2}}}f(\theta )$, where $f(\theta )=\frac{1+cos\theta }{sin\theta }[1+\frac{1}{3}{(\frac{1+cos\theta }{sin\theta })}^{2}]\cdot \frac{1}{2\sqrt{\pi }}$ is a geometric factor depending only on the contact angle *θ*. The variation of the 2D-island volume can be written, once corrected for the self-shrinking effect, as ${\rm{\Delta }}{A}_{2D}\cdot a$. ${A}_{droplet}$ (resp. ${A}_{2D}$) is the projected area of the droplet (resp. 2D island) measured on the LEEM image and *a* is the atomic height of the 2D island. The two values of volume change are equal for a contact angle of 43 ± 3 deg, which is equal to that of Au-Si droplets on Si (111)^31^. For the calculation we have considered that the Ge concentration follows the liquidus curve during the temperature change (+10 °C), that means a droplet volume variation of ≈0.15%.

### Droplet motion by dissolution of surface steps

We now consider the case of droplets that have nucleated at monoatomic steps on nominal or vicinal surfaces. A careful analysis of the droplet motion enables to describe the details of the step dissolution associated to motion parallel to the step or perpendicularly to it, i.e. across a terrace. In the following we will illustrate the various scenarios with Au/Si(111) or Au/Ge(111).

**Figure 7:**
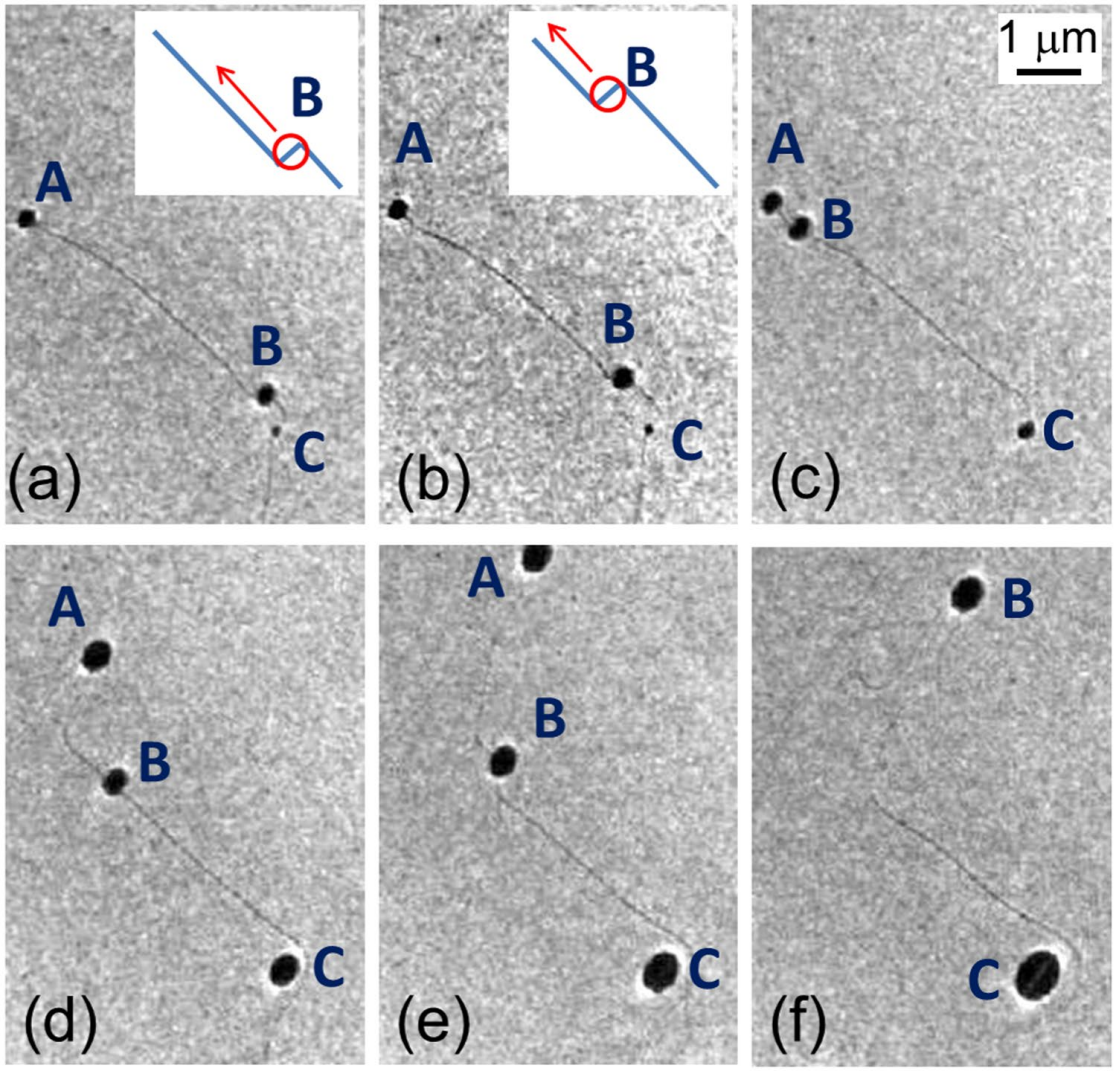
LEEM image of three droplets at a Ge(111) step (E = 2 eV, temperature = 750 K) during Au deposition (the total amount of Au on the substrate increases from 8.5 · 10^18^ to 14.0 · 10^18^ at · m^−2^). Droplet A creates a notch in the terrace, then moves perpendicularly to the step consuming it. Droplet B displaces along the step by kink consumption, then it is confined in a region where it changes the step curvature and moves normal to the step like droplet A. Droplet C grows on the spot. The insets in (**a**) and (**b**) show a sketch of the droplet motion due to kink consumption.

Figure 7 shows six images taken from a LEEM film (Supplementary Video S3) that illustrate the motion of growing droplets that have nucleated at a Ge monoatomic step. Because of the impinging Au flux, the droplets incorporate Au and thus have to dissolve Ge to keep their equilibrium concentration. Since the (111) surface is hard to dissolve, the droplets tend to incorporate Ge from the less bound sites. The droplet labelled A in Fig. 7 moves perpendicular to the step and thus nibbles the upper-terrace; this case is discussed later. The second droplet (labelled B in Fig. 7) moves very fast (300 nm/s) along the step and clearly nibbles it until it is locally pinned; then, as droplet A, it moves more slowly (15 nm/s) perpendicular to the step by dissolution of the upper-terrace. The third droplet (labelled C in Fig. 7) simply grows on the spot, probably pinned at a defect, and thus incorporates only the Ge atoms diffusing on the surface. Table 1 summarizes the behaviours of the three droplets. As a result, the adjacent step, which is in equilibrium with the gas of Ge adatoms, is consumed at distance and retracts towards the top-right part of the image. Still droplets in the middle of a terrace start to move very fast when they are reached by a moving droplet that displaces a step (see Supplementary Video S3). These kinds of droplets are therefore far from the equilibrium composition, as they do not have an easy way to dissolve Ge atoms, but when a step reaches them (dragged by another droplet or because of the surface rearrangement due to Ge diffusion), they quickly attain equilibrium. This very fast motion shows that the slow movement of droplets across terraces (like droplet A) is not limited by the dissolution kinetics. Besides, the projected area of still droplets grows faster than that of moving droplets but the volume increase due to Au incorporation should be the same. Thus, we deduce that still droplets are undersaturated in Ge and wet the substrate differently than moving droplets (they could also be solid Au particles).

**Table 1:** Behaviours of three droplets as described in the text.

Droplet	Motion	Velocity	Projected area
A	Perpendicular to the step	15 nm/s	Growing at 150 nm^2^ s^−1^
B	Along the step and later perpendicular to the step	300 nm/s and later 15 nm/s	Growing at 150 nm^2^ s^−1^
C	Does not move	0 nm/s	Growing at 800 nm^2^ s^−1^

Let us consider more carefully the droplet motion along a step (droplet B). The droplet motion is initiated by consumption of a kink (In this context we use kink as a general term, we do not only mean “monoatomic kink”), easier to dissolve than the (111) surface. Since the kink dissolution retracts the kink position, droplet B goes from C towards A by unzipping raws of atoms of the upper terrace (see insets of Fig. 7a and b). When arriving close to A, the droplet locally executes lateral small-amplitude oscillations before being in contact with the upper terrace on a large portion of its triple line. The droplet can now dissolve Ge only by moving across the terrace. This motion is much slower than that along a step because more atoms can be dissolved by the droplet with less displacement. A track is left behind the droplet, corresponding to the edges of the upper terrace. Notice that the track is less anisotropic than for Au/Si(111).

The droplets can also stochastically move without Au flux and without temperature change, because of Brownian diffusion and/or slight deviations from equilibrium. In this case the larger the local curvature is, the shorter the mean displacement around a given point is. This is discussed in Supplementary Discussion S4.

**Figure 8:**
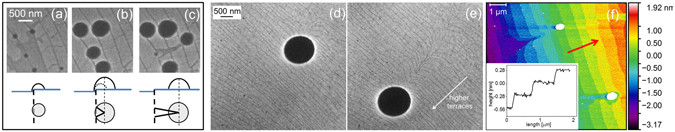
LEEM images (E = 2 eV) of droplets growing during Au deposition on Si(111); (**a**) Au islands nucleate at steps. The surface undergoes a $\sqrt{3}\times \sqrt{3}{{\rm{R30}}}^{\circ }$ (dark) → 6 × 6 (bright) phase transformation due to Au enrichment^25^; (**b**) the center of mass of the growing droplets displace towards the upper terrace; (**c**) a droplet has moved, leaving a track. A portion of the droplet remains at the defect where the droplet has nucleated. The total amount of Au on the substrate increases from 8.8 · 10^18^ to 40.7 · 10^18^ at · m^−2^; temperature = 660 K. Local miscut: 0.03°. Bottom: Side and top view sketches of the mechanism. The initial step position is reported in blue in the side view, whereas the notch formation is sketched in the top view (bottom images); (**d**) and (**e**) LEEM images (E = 1.8 eV, temperature = 690 K) of a large droplet growing during Au deposition from 3.7 10^19^ to 4.6 · 10^19^ at  · m^−2^ and climbing up (as verified by AFM) a vicinal surface, with formation of a set of notches. Local miscut: 0.07°. (**f**) AFM image showing droplets (white) that have moved and left traces on a (111) Si substrate. The droplets climb the terraces, as shown in the inset by the height profile taken along the red arrow. Local miscut: 0.03°.

When locally pinned at a step, a droplet may nibble the upper terrace and thus may move in a direction roughly normal to the step (see for instance droplet A in Fig. 7 for Au/Ge(111)). This is the typical behaviour observed for Au/Si(111). This case has been partially reported in ref. 25. Figure 8a shows a Si(111) surface with large terraces separated by monoatomic steps covered by a monolayer of Au with a LEED pattern corresponding to a 6 × 6 reconstructed 2D phase. After the completion of the 2D phase, going on with deposition, Au droplets nucleate at steps, then their volume increases due to Au incorporation by surface diffusion.

For Au/Si(111), contrary to the case of Au/Ge(111), only few droplets move along the steps. Figure 8 shows that, even if the droplets remain pinned at the steps, their center of mass displaces towards the upper-terrace. The Si consumption of the step by the droplet is evidenced by the track left behind (Fig. 8c) whose transitory darkening is due to a local $6\times 6\to \sqrt{3}\times \sqrt{3}{{\rm{R30}}}^{\circ }$ transformation (not reported LEED experiments), associated to local Au depletion that is quickly healed by surface diffusion (so that the initial 6 × 6 is recovered).

The mechanism we propose is sketched in Fig. 8 where side and top views show the droplet motion with respect to the initial step position and the step consumption under the droplet. In the initial stage, the step dissolution forms an interfacial notch which cannot be seen but the center of mass of the droplet displaces with the growing notch. The substrate can be easily dissolved at the apex of the notch, therefore a growing droplet will tend to dissolve Si from it, increasing the notch size and moving to stay pinned to it. Notice that the droplet is not pinned where the step has already been consumed. The notch left behind the droplet forms a track.

From crystallographic arguments, the notch can be limited by <110> directions, where the substrate atoms are denser and therefore more bound than in other directions. As there are three equivalent <110> directions, the track may present triangular features.

The notches formed by the moving droplets are easier to visualize when the droplet diameter is larger than the interstep distance. In this case the droplets stride over many steps, so that the droplets move on the vicinal surface by step consumption leaving notches that collectively form wakes behind the moving droplets (see Fig. 8d and e). The droplets climb up the steps as shown in Fig. 8f (the inset shows a profile taken along the red arrow). Actually, by selectively etching Au with KI/I_2_, we find a shallow hole beneath the droplet (Fig. 3) which is not visible in the track of Fig. 8. This means that the moving droplets must drag their own hole. Such a behaviour implies that Si dissolution from the advancing front partially leads to Si recrystallization at the back to fill up the hole^5^.

### Au/Si(111) versus Au/Ge(111)

For both systems, Au/Si(111) and Au/Ge(111), the substrate dissolution takes place by step consumption that corresponds to the easier dissolution sites. This dissolution leads to a spontaneous droplet motion. However there are important differences between the two systems. For Au/Ge(111), most of the droplets essentially move along the steps, and consume step atoms. Because of defects, a droplet can change the local curvature of a step, and create an indentation where it pins. This is then used to nibble the upper terrace. For Au/Si(111) most of the droplets directly nibble the upper terrace with no or only very limited motion along the steps. These differences may be interpreted by considering the energetics of Si and Ge crystals as well as their anisotropic properties. If we assume that the kink energy is a fraction of the cohesion energy (384 kJ mole^−1^ for Si and 334 kJ mole^−1^ for Ge) and if we use 320 meV^32^ as the kink energy on a Si(111) surface, we find that the Ge kink energy should be 278 meV. Therefore a monoatomic step on Ge(111) should exhibit, at equilibrium, twice more kinks than a Si step ($\frac{exp(0.320/(kT))}{exp(0.278/(kT))}\approx 2$ for a typical temperature of 700 K). Besides, since the cohesion energy of Ge is lower than that of Si, dissolution is expected to be easier on Ge than on Si. Furthermore we have observed that the notches left by the droplets after step consumption have straight, well defined edges for Au/Si(111), whereas they are more rounded for Au/Ge(111). This suggests that the dissolution anisotropy is stronger for Si than for Ge. Furthermore, as shown in the phase diagrams of Fig. 1, Au can dissolve Ge also in the solid state, while it must form a liquid alloy to dissolve Si. This could be at the origin of a retarded motion of Au on Si with respect to Ge: a solid Au island nucleates and grows on Si before dissolving the substrate to become liquid and therefore moving. We also notice that much larger terraces have been obtained on Ge than on Si, but this should not change the droplet motion behaviours.

## Droplet motion associated to easy dissolution on ($11{\boldsymbol{\ell}}$) substrates

For ($11\ell$) surfaces (with $\ell \ne 1$), which are easier to dissolve than the (111) surfaces, the holes formed by dissolution are less symmetric than on (001), and form polyedric shapes limited by the dense facets.

### Au/Si(110)

**Figure 9:**
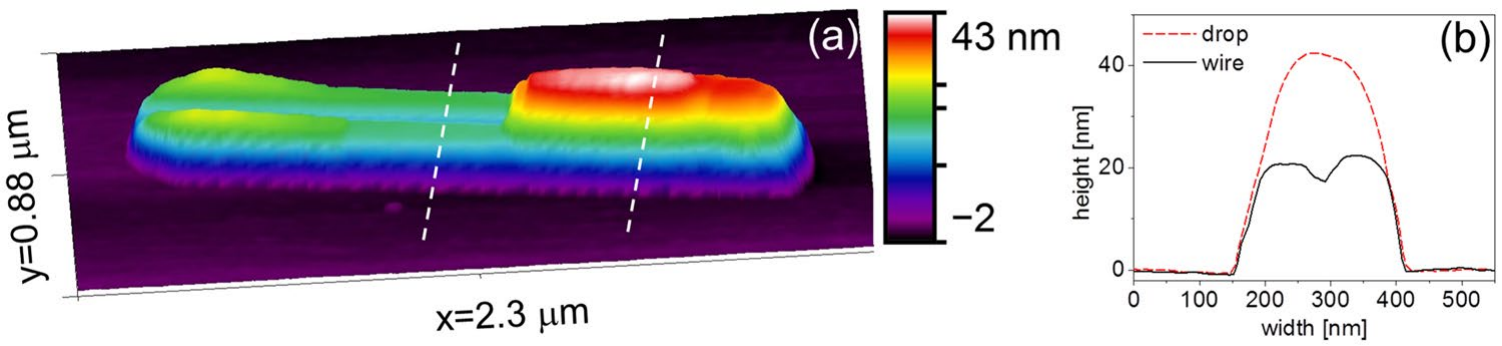
(**a**) 3D AFM image taken at room temperature of a Au-Si droplet on Si(110) which has formed a Si nanowire at 720 K after deposition of ~3 · 10^19^ at · m^−2^ of Au. (**b**) The height profiles taken along the white dashed lines of (**a**) show that the droplet is higher than the wire.

On Si(110), the droplets elongate keeping constant width, and move along the [1–10] axis in either direction according to the droplet. The moving islands fabricate an elongated track behind them (see Fig. 9). AFM profiles recorded before and after Au selective dissolution shows that the droplets form deep asymmetric holes underneath, and the tracks behind them are not trenches due to Si dissolution but nanowires due to Si expelled from the moving droplets (Fig. 9b). In ref. 28 the wires are interpreted as produced by a convection current in the droplets and thus a difference of Si concentration between the front and the back of a droplet.

A droplet grows steadily in one direction and jumps from time to time. The jumps do not affect the droplet length, which increases linearly with time. We have measured by AFM that the holes under the droplets are deeper than the emerged part of the droplets. At the temperature of our experiments the droplets should not contain more than 40% Si (according to the equilibrium phase diagrams of Fig. 1). Therefore the droplets dissolve more Si than necessary and the excess of Si must be redeposited somewhere.

**Figure 10:**
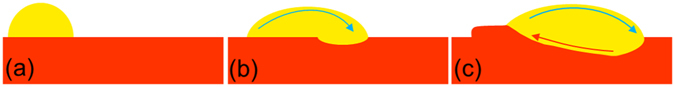
Mechanism of droplet motion and wire formation on Si(110) during Au deposition. (**a**) A Au island has nucleated on the substrate; (**b**) the island is pinned at one edge and, growing, it elongates at the other edge. The elongation can be considered as a Au flux towards the growing edge. (**c**) Si is dissolved at the growing edge, where more Au arrives, and moves opposite to the Au flux towards the pinned edge at the back. Si is accumulated at the back, and forms a wire.

A mechanism is proposed in Fig. 10. The different steps of the mechanism take place simultaneously, but for the sake of clarity they are separated in the discussion below: (**1**) As Au is deposited on the substrate, the droplet grows and dissolves the substrate to reach the equilibrium composition. Its shape is elongated because the dissolution of the interface is limited by {111} facets (see Fig. 3i) and the droplet-substrate-atmosphere triple line corresponds to the edges of the hole in the substrate. (**2**) The droplet grows by Au incorporation. Depinning is difficult from the long lateral edges, while it is easier from one of the corners. This becomes the advancing front. The elongation of the droplet implies a Au flux towards the growing edge. To compensate the Au supersaturation, the Si substrate is dissolved at the advancing front, and a flux of Si opposite to the Au flux is generated into the droplet. (**3**) Si is accumulated and redeposited at the back edge, where the concentration of Au is lower and it forms a nanowire. The droplet jumps to pin to the edge of the redeposited Si.

### Au/Si(113)

**Figure 11:**
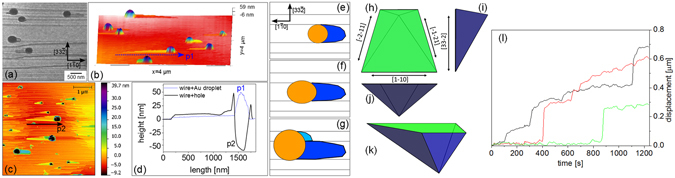
(**a**) LEEM image (E = 2.5 eV, temperature = 710 K) of Au droplets moving and forming a wire on Si(113) at 710 K after deposition of ~3   10^19^ at · m^−2^ of Au; (**b**) 3D AFM image of Au droplets which have formed nanowires on Si(113); (**c**) AFM image after selective etching of Au, taken in a different place than (**b**). Holes are left at the place of droplets; (**d**) height profiles along the lines p1 and p2 drawn in (**b**) and (**c**); (**e**–**g**) schematics of the formation of a double nanowire, as detailed in the text; (**h**–**k**) top, side and tilted views of a hole limited by {111} facets, as calculated with the KrystalShaper software^43^; (l) position of three Au-Si droplets on Si(113) as a function of time, during Au deposition at 770 K. The Au flux is 1.8 · 10^16^ at · m^−2^ s^−1^.

Let us now consider the case of the Si(113) surface that, upon Au deposition, forms a hill-and-valley morphology in the [1–10] direction (period 70 ± 30 nm, amplitude 2 ± 1 nm, see Fig. 11a). LEEM films show that the droplets move along the [1–10] direction (see Fig. 11a and Supplementary Video S5). Growing droplets elongate in the grooves formed by the hill-and-valley morphology and are weakly pinned on edges parallel to the [1–10] direction that also correspond to the surface projection of the {111} facets formed by substrate dissolution. The growing droplets, since only weakly pinned, periodically de-pin from one of the lateral ridges and thus enlarge. The droplet shape is thus more isotropic than on Si(110), but actually results from successive lateral enlargements. As long as a growing droplet is confined, it forms an elongated nanowire at its back by a mechanism similar to the one we describe for Au/Si(110). However the depinning events enlarge regularly the droplet width and thus the nanowire width. Therefore at the end of the experiment we obtain more or less spherical droplets followed by a triangular track (see the mechanism sketched in Fig. 11e–g). The hole in the substrate is more isotropic than for Si(110): As shown in Fig. 11h–k, it is symmetric with respect to the (1–10) plane but asymmetric with respect to the (33-2) plane. The wires grow much slower and the droplets move less. Besides, the asymmetry with respect to the [1–10] direction is coherent with the triangular track extending laterally always in the same direction (toward the upper part of Fig. 11a and c, for all droplets).

Droplets growing on Si(113) move by successive jumps, as a result of the formation of a nanowire. The motion kinetics is very slow if compared to other substrate orientations and is therefore difficult to study because requires long experiments. The displacement as a function of time of three droplets is shown in Fig. 11l.

## Discussion

The droplet motion is due to the chemical etching of the substrate. However we show that the exact scenario depends on the surface orientation and on the local morphology of the surface. In case of confined droplets, the spontaneous motion is associated to the formation of a nanowire behind every moving droplet. The phase diagrams of nanoscale systems are size-dependent^33, 34^. However we exclude that the mechanisms observed are due to a nano-scale concentration change, because the mechanisms described are observed also for droplets much larger than 200 nm (which is the limit for a size effect on the phase diagram^34^). We infer that the nanowire growth results from a convection flux inside the droplet. Indeed, the droplet growth is constrained by the underlying asymmetric hole that confines the droplet. Such a geometrical constraint, combined with Au incorporation from the capture area of the droplet leads to a strong asymmetry of the Au flux towards the moving front and thus a strong asymmetry of the Si flux (in the opposite direction) inside the droplet. The existence of asymmetric fluxes might be checked by analyzing the mass and heat transfer inside a constrained growing droplet. Formally, the hydrodynamic behaviour and the associated concentration evolution inside a spreading and moving droplet should be numerically simulated from the resolution of Navier Stokes equation (as done in refs 35–39) but including an additional reaction term^40, 41^ as well as the anisotropy of etching, which leads to the strong droplet elongation. Writing down and solving such equations is a tremendous challenge which is out of the scope of this paper. However, a short discussion (see Supplementary Discussion S6) may be initiated from simple flux balance arguments to show that the nanowire formation is associated to an over-etching.

## Conclusions

We have shown that, due to anisotropic etching, a growing droplet forming an alloy with the substrate moves differently according to the orientation of the substrate. We have explained different scenarios of droplet motion for two similar systems: Au/Si and Au/Ge. The shape of the pit under the droplets is controlled by the underlying substrate symmetry. On easy-to-dissolve substrates, like (001) surfaces, a growing droplet simply dissolves the underlying substrate on the spot. On hard-to-dissolve surfaces as the (111), growing droplets use monoatomic steps or 2D island edges as easier sites of dissolution and thus spontaneously move on the surface following the step edges by kink dissolution or by 2D island consumption. With a careful analysis of various emblematic cases observed for Au/Si(111) and Au/Ge(111), we describe the mechanisms that lead to a subtle interplay between surface defects (steps, 2D islands) and droplets during their growth at constant temperature or during a temperature change, in absence of any external flux. The droplet velocity depends on the relative size of the droplet with respect to the terrace width^25^ and thus depends on the amount of Au deposited and on the number of nucleated droplets (influenced by the flux rate, the temperature and the number of defects). Moving droplets and their underlying hole generally displace at once. The droplet motion can thus be described by substrate dissolution at the front of the droplet and substrate recrystallization at the rear. Table 2 summarizes the findings.

**Table 2:** Scenarios of the behaviours of Au-rich droplets on different orientations of Si and Ge.

(001)	no motion/deep substrate dissolution
(111)	motion/dissolution of mono-atomic steps
(110)/(113)	elongation/motion/formation of nanowires

The present systematic study of self-propelled droplet motion induced by interfacial reactivity brings new insights and perspectives useful for a wide scientific community since (i) anisotropic etching is a key tool used in many Si or Ge-based device manufacture, (ii) Au droplets are widely used as catalysts for Si and Ge nanowires, their shape and position have to be carefully controlled, so that it is important to understand the droplet motion induced by reactivity, and (iii) the formation of in-plane Si nanowires and of droplet-hole pairs could be useful to pattern semiconductor substrates.

## Methods

We have used B-doped silicon wafers with 0.01° nominal miscut and Sb-doped germanium wafers with 0.5° nominal miscut. The substrates have been cleaned with acetone, ethanol then distilled water in ultrasonic bath before introduction into the UHV chamber (basic pressure 5 · 10^−10^ mbar). Si surfaces have thus been cleaned in UHV by flashing above 1400 K, to remove the surface oxide, followed by thermal annealing at 1000 K to get reconstructed surfaces with regular terraces.

Ge surfaces have been obtained by repeated cycles of ion bombardment (Ar^+^, E = 1k eV, I = 8 μ A) and annealing (1000 K). Finally the Ge substrates have been flashed close to the melting point (1211 K) to get extended terraces. The quality of the so-obtained surfaces is confirmed by the observation of LEED patterns characteristic of the well-known surface reconstructions classically observed for the different surface orientations. Moreover, LEEM imaging enables to check the absence of local impurities over a length scale of hundreds of micrometers.

Au (99.999% pure) has been deposited by evaporation-condensation from a MBE-Komponenten effusion cell at *T* =1480 K _E_ (flux ~10^16^ at · m^−2^ s^−1^).

We have studied *in situ* and in real time, by Low Energy Electron Microscopy (LEEM-Elmitech III), the behaviour of Au droplets on Si and Ge substrates with different crystallographic orientations. LEEM images have been taken with a frequency of 0.5–1 Hz and an electron energy in the range 1–10 eV.

The details of the droplet formation have been described elsewhere^25, 42^, and are only summarized here: Au deposition starts with the formation of a 2D phase which completely fills the surface (one monolayer) before the formation of 3D islands. For *T* > *T*_E_ the 3D islands are solid whereas for *T* > *T*_E_ their equilibrium state is a liquid alloy resulting from substrate dissolution.

At the end of the LEEM experiments, the samples are quenched and thus the droplets crystallize. Their morphology is characterized *ex-situ* by Atomic Force Microscopy (AFM) in air, using a Park XE-100 in tapping mode. In order to study the morphology of the etch pits formed under the droplets, we also use a standard KI/I_2_ solution to selectively etch Au. Energy Dispersive X-ray analysis was performed with 15 kV electron energy in a SEM Jeol JSM-6320F equipped with a Quantax analyzer to measure the Si and Au contents. No Au was detected after KI etching. The Si dissolved when the droplets are liquid and that is redeposited during solidification could mislead the measurements of the size of the hole beneath the reactive droplets. However Si deposits at the edges of the holes (see profiles of Fig. 3), and also accounting for this effect, the difference of hole depth in the different orientations is meaningful. In the nanowires, as shown in Supplementary Figure S7, no Au was detected and therefore only a small amount of Au, below the EDX threshold detection limit, could be present; nevertheless the only possible Si and Ge rich stable phases, according to the bulk phase diagrams, are almost pure Si and Ge. If the wires were constituted of known metastable phases^29^, they should have a Au content of at least 35 at.%.

The calculations of the etch pit shape are performed with the KrystalShaper software^43^.

## Electronic supplementary material

Supplementary information

Au-Ge droplets decreasing temperature

Au-Ge droplet interacting with 2D Ge islands

Au-Ge droplets moving on Ge(111)

Au-Si droplets moving on Si(113)
