# Supplementary material for: Surface-dependent scenarios for dissolution-driven motion of growing droplets
Source: Sci Rep. 2017 Apr 19;7:902. doi: 10.1038/s41598-017-00886-2 (PMC5430430; doi:10.1038/s41598-017-00886-2)
Supplement: Supplementary file 1 — Supplementary information [file 41598_2017_886_MOESM1_ESM.pdf]

Surface-dependent scenarios for dissolution-driven motion of growing droplets

Stefano Curiotto[1,*], Frédéric Leroy[1], Fabien Cheynis[1], and Pierre Müller[1]

[1]Aix Marseille Univ, CNRS, CINaM, Marseille, France
[*]curiotto@cinam.univ-mrs.fr

List of supplementary material

S1: film of two Au-Ge droplets pinned at a 2D island on Ge(111). Decreasing the temperature from 750K to 660K, with intermediate steps at 720K and 690K, the droplets release Ge to the 2D islands and move. The width of the window is 8.9 μm. The film is accelerated; the original duration is 914 s. The LEEM electron energy is 2 eV.
S2: film of a Au-Ge droplet that consumes 2D Ge islands increasing the temperature from 890K to 900K. The droplet moves following the edges of the 2D islands. The width of the window is 3.6 μm. The film is accelerated; the original duration is 109 s. The LEEM electron energy is 2 eV.
S3: film of Au-Ge droplets nucleating and interacting with a Ge(111) step during Au deposition at 750K, as described in the text. The width of the window is 10.7 μm. The film is accelerated; the original duration is 1243 s. The LEEM electron energy is 2 eV. The black spot on the top right part is a defect of the channel-plate.
S4: Description and modelling of the stochastic motion of droplets at a step, according to the step curvature.
S5: film of Au-Si droplets nucleating and interacting on a Si(113) substrate during Au deposition at 710K, as described in the text. The width of the window is 4.4 μm. The film is accelerated; the original duration is 1249 s. The LEEM electron energy is 2.5 eV.
S6: Discussion of the droplet motion and nanowire formation on (110) surfaces, using simple mass-conservation arguments.
S7: Figure of the EDX signal originated from a droplet, a wire and the substrate for Au/Si(110).

S4 - Step-curvature effect on the droplet motion

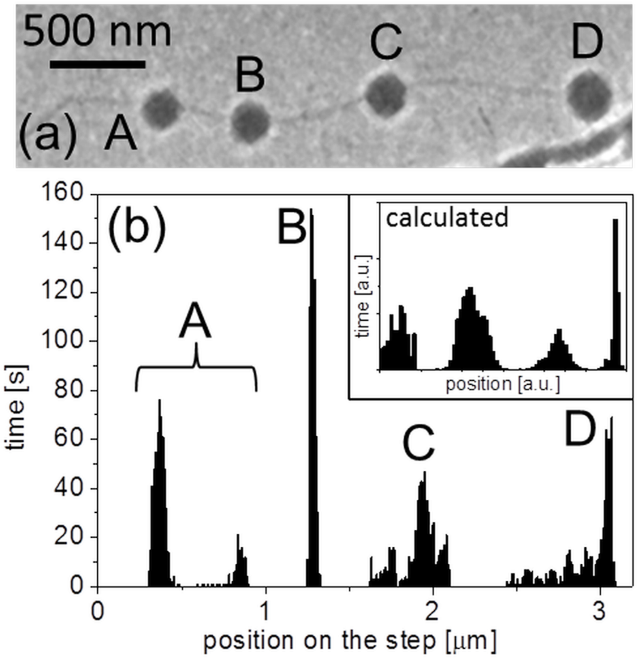

Figure 1: (a) LEEM image (E=2 eV) of four droplets moving along a slightly curved step; (b) Distribution of the droplet positions along the step. Notice that droplet A has explored two positions since has jumped from one to the other. The inset shows the calculated distribution of the droplet positions in a simple model (with $\alpha = 0.01$).

Figure S1 shows four droplets on a locally curved step. We have measured the position of the droplets on the step as a function of time. The histogram of these positions shows that the droplets stochastically move along the step but the distance explored by each droplet depends on its position on the step. More precisely, the larger the local curvature is, the shorter the mean displacement around a given point is. This means that the local motion actually depends on the local number of sites where the droplet can be temporarily pinned. Notice that, during the measurements, the step shape slightly changes with time because the droplets nibble the step.

This droplet behaviour can be easily reproduced by a simple phenomenological model in which we simulate the 1D random motion of a single droplet along a weakly curved step with the simple assumption that the length of a unit jump a per unit time depends on the local curvature C, as $a = a_o\,(1 - \alpha C)$ where α is a phenomenological parameter and a_o an atomic unit length. The experimental step is simulated by a polynomial function so that the local curvatures can be

easily calculated. In the calculation we consider a single droplet that explore the whole step. Furthermore we assume that when the droplet reaches an extremity of the step it re-enters at the other extremity. The calculated histograms of the droplet positions (Figure S1) show that a droplet stays longer in the curved regions, from which it can jump to another curved zone. In the experiments, the droplets locally modify the curvature by nibbling the step, which is not the case in the simulation so that the agreement is not perfect, especially in terms of mean displacement, however we find the four positions where the droplets are more or less trapped.

S6 - Drop motion discussion

The droplet shape can be modeled as a rectangular box (width L, emerging height H, submerged depth h, see the figure below), whose advancing front moves with a velocity V_f equal to the local dissolution rate. Let us write the Si flux balance as $xFA = J_f - J_b$ where F is the Au incoming flux, A the droplet capture area and thus xFA the Si amount that must be dissolved to reach the droplet equilibrium composition. The droplet can form a nanowire only if the Si back flux J_b is positive and therefore if the Si front flux $J_f > xFA$. The Si front flux can be written $J_f = V_f(C_s - C_l)hL$ where C_s and C_l are concentrations of Si atoms per unit volume in the solid and in the liquid respectively. We take $(C_s - C_l) \approx C_s$, as the droplet volume increase is mainly due to Au. For a droplet spreading at the front, there is a Au flux $J_{Au} = FA = V_f \cdot H \cdot L \cdot C_{Au}$, where C_{Au} is the concentration of Au in the advancing liquid. The Si flux at the front necessary to reach equilibrium can be written as a fraction of this Au flux, $x \cdot J_{Au}$. J_b is positive if $J_f > x \cdot J_{Au}$, or $\frac{h}{H} > x \cdot \frac{C_{Au}}{C_s}$. For Au/Si, at the temperatures of our experiments, $0.25 < x < 0.7$. On Si(110) we have found, after solidification, $h/H \approx 1.3 \pm 0.4$, which is much larger than $x \cdot \frac{C_{Au}}{C_s}$ (taking $C_{Au} = 5.3 \cdot 10^{28} at \cdot m^3$ and $C_s = 5 \cdot 10^{28} at \cdot m^3$) and therefore J_b is positive. With a Si flux towards the back of the droplet, Si is accumulated resulting in the formation of a nanowire.

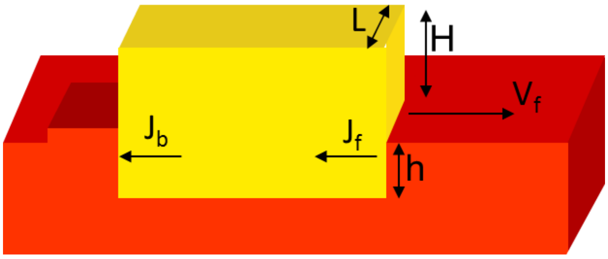

Figure 2: A droplet on a substrate is schematically drawn as a rectangle with length L, emerging height H, submerged depth h.

S7 - EDX analysis on Au/Si(110)

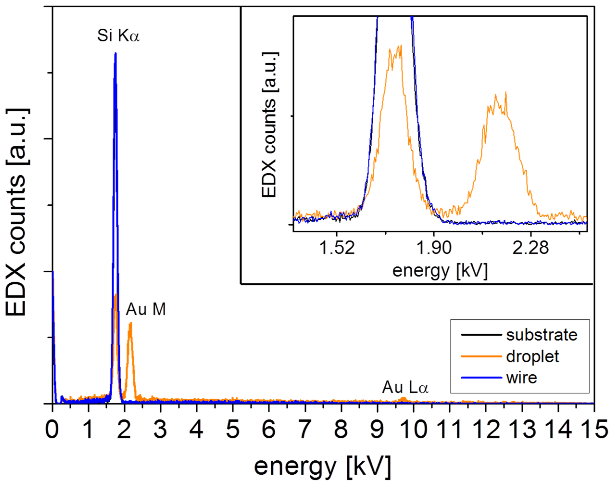

Figure 3: EDX analysis of a Au-Si droplet (yellow), a wire formed by the droplet (blue), and the (110) Si substrate (black). The sample has been transferred from the LEEM to the SEM in air. The wire spectrum is very close to that of the substrate. As shown in the magnified portion of the spectrum (inset), no Au is detected in the wire. The spectrum of the droplet shows a Si peak because the droplet has dissolved Si and because part of the signal comes from the substrate under the droplet.
